# Resistance to chemoimmunotherapy in non-small-cell lung cancer

**DOI:** 10.20517/cdr.2020.09

**Published:** 2020-07-12

**Authors:** Maximilian Johannes Hochmair

**Affiliations:** Respiratory Oncology Unit, Vienna North Hospital - Klinik Floridsdorf, Vienna 1210, Austria.

**Keywords:** Lung cancer, chemotherapy, immunotherapy combination, pembrolizumab, resistance

## Abstract

Recent clinical trials evaluating the combination of chemotherapy with immune checkpoint inhibition for the primary treatment of lung cancer showed increased progression-free and overall survival compared with chemotherapy alone. However, the combination of these two modalities is less than additive and the mechanisms of resistance to this therapeutic intervention are discussed here. So far, the conventional biomarkers for immunotherapy, namely programmed death-ligand 1 expression or tumor mutational burden are poor predictors of the efficacy of immunochemotherapy, and the optimal sequence of chemotherapy and immunotherapy has yet to be defined.

## Introduction

Lung cancer shows the highest incidence of tumor-related mortality and accounts for approximately 2 million new cases per year worldwide^[1]^. The 5-year overall survival (OS) rate for non-small-cell lung cancer (NSCLC) remains poor, from 70% in patients with stage IB disease to 0-10% in patients with stage IV disease^[2]^. Advances in targeted therapy and immunotherapy have resulted in a significant improvement which is, however, experienced only by a minor fraction of all lung cancer patients. The most common targets of precision cancer medicine for this tumor type are epidermal growth factor receptor mutations, which are present in 15% of patients, rearrangements in the anaplastic lymphoma kinase gene as driver for approximately 5% of patients, and ROS1 rearrangements identified in 1%-2% of patients, besides other rare mutated driver kinases amenable to treatment with specific inhibitors. Additionally, immunotherapy is now applied frequently for lung cancer patients in a primary or secondary setting, but again, marked benefits are limited to approximately 15% of patients, mostly characterized by high immune checkpoint molecule expression or elevated tumor mutational burden^[3]^. Studies have demonstrated a median OS of 20.0 and 30.0 months with pembrolizumab for locally advanced/metastatic patients exhibiting programmed death-ligand 1 (PD-L1) expression equal to or exceeding 50% of tumor cells^[3]^. Although targeted therapies have offered new treatment options for NSCLC patients with specific driver mutations, these therapies are not helpful in the care of patients lacking such alterations, who account for the majority of patients. Therefore, the greater fraction of the lung cancer patients has still to be treated with cytotoxic chemotherapy. Response rates and durations of chemotherapeutic regimens are rather low, and combinations with immunostimulating modalities have been implemented to improve outcomes.

### Chemotherapy for NSCLC

For the majority of patients diagnosed with advanced NSCLC, cytotoxic chemotherapy still remains the standard of care. Adjuvant therapy for NSCLC consists of platinum-based drug regimens and is applied for patients with stage II and IIIA disease after surgical resection. Carboplatin or cisplatin are combined with gemcitabine, vinorelbine, or taxanes (paclitaxel or docetaxel) and these regimens result in a median OS of 10-12 months for patients with non-squamous histology^[4]^. The standard pemetrexed combination is better tolerated compared to the other doublets.

Platinum-based chemotherapy accomplishes a modest extension of survival and addition of a third chemotherapeutic shows minor improvements^[4]^. Drug resistance to platinum-based doublets can be explained by low drug influx or increased efflux through the cell membrane, lack of intracellular activation, and increased drug inactivation or detoxification. For example, platinum agents are detoxified by coupling to glutathione and excretion of the conjugates. Furthermore, the cytotoxicity of these drugs depends on the cellular ability to repair DNA damage and alteration of the apoptosis signaling cascades. In summary, the efficacy of chemotherapy in advanced NSCLC is rather low and the situation is characterized by high chemoresistance and poor OS. No predictive biomarker is currently available for classical cytotoxic chemotherapy contrary to targeted and immune therapies^[5]^.

### Immunotherapy for NSCLC

The reactivation of anticancer immunity by antibodies targeting checkpoint inhibitor molecules such as PD-1/PD-L1 and CTLA4 in cancer patients has led to new therapeutic options completely replacing chemotherapy in suitable patients. Single-agent immune checkpoint inhibitors are now administered for advanced NSCLC in both first-line therapy and second-line after failure of the initial chemotherapy. Factors qualifying NSCLC patients for immunotherapy are extended disease, absence of driver mutations and higher expression levels of PD-L1. In first-line therapy, PD-L1 expression of ≥ 50% makes patients candidates for monotherapy with the anti-PD-1 antibody pembrolizumab and others. Whereas an objective response rate (ORR) of 45% is observed in PD-L1 of 50% or more, this ORR drops to 27% in patients with PD-L1 of 1% or more^[6]^. However, the significance of the detection of PD-L1 in cancer cells by immunohistochemical analysis as biomarker is difficult to assess due to a significant intratumor heterogeneity and the lack of consensus as to the level of PD-L1 expression that discriminates positive *vs.* negative immunotherapy results (ranging 1%-50% expression)^[7]^.

A phase I study of pembrolizumab in patients with advanced NSCLC has shown high efficacy, revealing a median OS of 22.3 months in primary patients and a median OS of 34.9 months in patients whose PD-L1 tumor proportion score (TPS) is R 50%^[8,9]^. This superiority was confirmed by a phase II and III study, demonstrating that treatment with pembrolizumab prolonged OS by 2-4 months in PD-L1-positive (TPS R 1%) NSCLC patients who progressed after platinum-based chemotherapy *vs.* standard-of-care treatment^[10]^. Several phase II/III studies compared second-line treatment with PD-1 antibody nivolumab *vs.* docetaxel and reported a doubling of OS (17.0 superior to 8.0 months) in response to this immunotherapy, and similarly, atezolizumab was found to be superior to docetaxel (13.8 *vs*. 9.6 months) in second-line treatment. However, the population of patients achieving a marked response that might result in long-term survival was found to be limited to a minority of approximately 20%^[11-13]^. Neither analysis of PD-L1 nor of tumor mutational burden constitutes perfect biomarkers for the response to immunotherapy, and positive results of immune checkpoint inhibitors may be seen in some marker-negative patients. Thus, most NSCLC cases do not benefit from immunotherapy, and for many responding patients, the effect is transitory. The mechanisms of resistance to checkpoint inhibition are multifaceted and an area of ongoing investigation.

### Immunochemotherapy for NSCLC

Patients with PD-L1 expression ≥ 50% are typically treated with single-agent anti-PD-1 antibody pembrolizumab but some of these patients with rapidly progressing or extensive tumors may profit from a combination of a platinum-doublet chemotherapy and pembrolizumab^[14]^. This combination therapy has proved to be more effective than chemotherapy alone for patients with metastatic NSCLC independently of their PD-L1 expression. The addition of pembrolizumab to the platinum doublet was not associated with increased grade 3 or 4 adverse events (AEs) compared with chemotherapy alone^[14]^. Regarding immune checkpoint-directed antibodies, similar results were obtained for chemotherapy combinations employing atezolizumab or nivolumab and are to be expected for other antibodies with the same specificity.

The approval of this type of chemotherapy - immunotherapy combination is based on the phase III KEYNOTE-189 trial which randomized 616 patients with advanced, PD-L1-unselected, non-squamous NSCLC in a 2:1 ratio to chemotherapy (cisplatin/carboplatin with pemetrexed) with or without pembrolizumab^[3,15,16]^. The pembrolizumab antibody was administered as 200 mg intravenous infusion every three weeks. Immunochemotherapy improved 12-month OS relative to chemotherapy alone [69% *vs*. 49%; hazard ratio (HR) for death 0.49]. These improvements were detectable in all PD-L1 categories, with greatest effects in PD-L1-positive tumors. PFS was also prolonged with the supplementation of pembrolizumab (8.8 *vs*. 4.9 months; HR for disease progression or death, 0.52) corresponding with an improvement of ORR (48% *vs*. 19%). The rate of severe AEs (≥ grade 3) was not significantly different for the pembrolizumab-combination and placebo groups (67% *vs*. 66%). These results corroborated findings of the earlier phase II KEYNOTE-021 trial, which had randomized 123 stage III B or IV non-squamous NSCLC patients in a 1:1 ratio to chemotherapy with or without pembrolizumab and reported improved ORR (55% *vs*. 29%) and median PFS (13.0 *vs*. 8.9 months)^[16]^. Atezolizumab with carboplatin and taxane-based regimens are also appropriate and have regulatory approval in this setting as well^[17-19]^.

In conclusion, the goal of combing immune-checkpoint inhibitors and chemotherapy to achieve additive or synergistic efficacy has been verified but questions remain. It is still not clear whether patients with high PD-L1 expression may benefit from immunochemotherapy since trials evaluating chemotherapy plus pembrolizumab *vs.* pembrolizumab alone are lacking. Cross-trial comparisons of KEYNOTE-024 and the PD-L1-high subgroup of KEYNOTE-189 indicate comparable outcomes between pembrolizumab combination therapy and pembrolizumab alone for PD-L1-high tumors (12-month OS rate of approximately 70% in both trials). The KEYNOTE-042 trial compared pembrolizumab monotherapy *vs.* platinum-based chemotherapy as first-line therapy for advanced NSCLC and a PD-L1 ≥ 1%^[20]^. KEYNOTE-407 evaluated chemotherapy (carboplatin-paclitaxel-nab-paclitaxel) with or without pembrolizumab for squamous NSCLC and demonstrated significant overall and PFS improvement with immunochemotherapy^[21]^. The clinical efficacy of the rechallenge with immune-checkpoint agents either as monotherapy or in combination with chemotherapy for patients having received first-line immunochemotherapy is unknown. Immunotherapy is costly and a comparison of the KEYNOTE-189 trial data between cost and quality-adjusted life years proved that immunochemotherapy is not cost-effective for the treatment of NSCLC compared with chemotherapy alone^[22]^.

### Chemotherapy and the immune system

Effects of chemotherapeutic drugs have been studied incompletely and have been regarded as mainly immunosuppressive^[23,24]^. In contrast to these opinions and expectations preclinical studies demonstrated that chemotherapeutic agents can stimulate the immune system and increase antitumor immunity as part of the potency of these drugs^[25,26]^. Accordingly, early clinical investigations revealed superior antitumor activity for a combination of chemotherapy and PD-1/PD-L1-directed antibodies in first-line treatment for advanced NSCLC^[27-30]^. Chemotherapy enhances antitumor immunity mainly by induction of immunogenic cell death and remodeling of the immunosuppressive tumor microenvironment^[31]^. Chemotherapeutic agents that increase antitumor immune responses by various mechanisms include drugs such as cisplatin, carboplatin, taxanes and pemetrexed used in NSCLC^[32]^. Chemotherapy seems to stimulate CD8^+^ T-cells, increase the mutational load, and the diversity of neoantigens as well as the maturation of antigen-presenting cells (APCs). Furthermore, PD-L1 expression on tumor cells becomes upregulated, and tumor antigen presentation via MHC class I is facilitated. Concurrently, immunosuppressive cells including regulatory T-cells (Tregs) and myeloid-derived suppressor cells are eliminated^[33-36]^.

This removal of immunosuppressive Tregs, MDSCs and a reduction of PD-1 ligand expression results in tumor vaccination which is potentiated by the delivery of tumor antigens, upregulation of MHC I and activation of APCs^[37-42]^. Additionally, the infiltration of T-cells into the tumor is increased by disruption of tumor stroma and normalization of the tumor vessel supply in combination with an upregulation of adhesion molecules of the tumor vasculature^[43]^. T-cell cytotoxicity against tumor cells is further enhanced by increased tumor Fas expression, Th1 cytokine production and increased CTL avidity, which eventually leads to the promotion of long-term memory T cells^[44]^. By these mechanisms, platinum-based chemotherapy promotes tumor-specific immunity, and the combination with immune checkpoint inhibitors may mount efficient effector T cell attacks.

## Resistance to immunochemotherapy

Despite the unique long-lasting response with checkpoint inhibitors, the majority of patients do not benefit due to primary resistance or relapse after an initial response, showing acquired resistance. Low expression of neoantigens, impaired antigen presentation, deficient interferon-γ signaling, and T cell exclusion as well are designated as tumor cell-intrinsic resistance factors. Tumor cell-extrinsic factors comprise inhibitory checkpoint molecules different from PD-1/PD-L1, immunosuppression by MDSCs and Tregs as well as deficient specific antitumor T cell responses^[45]^. The most important reason for refractoriness to immune checkpoint therapy is the absence of tumor neoantigens that can be recognized by T cells^[46]^. At a second step of anticancer immunity, suitable tumor antigens are expressed but their presentation in combination with MHC on the tumor cell surface fails due to alterations in the antigen presenting mechanism such as in proteasome subunits, transporters associated with antigen processing, beta-2-microglobulin or MHC itself^[47]^. Furthermore, immune cell infiltration or function within the tumor microenvironment is prevented by increased PI3K signaling, expression of the WNT/β-catenin signaling pathway and the loss of interferon-γ signaling^[47]^.

For immunochemotherapy, the prerequisite for mounting an efficient anticancer immune attack may be a certain degree of tumor cell damage and the expression or release of neoantigens into the circulation. At present, it is not clear whether direct chemotherapy-triggered effects on tumor cells and an increase of neoantigens or indirect effects on the immune system such as elimination of immunosuppressive cell populations are the decisive mechanisms of immunochemotherapy^[42,44]^. Still, the actual mechanisms of resistance to immunochemotherapy need to be studied *in vivo* in patients to identify the most important underlying cellular events. Such an investigation has to involve the assessment of longitudinal tumor samples throughout the course of treatment. Key findings in a recent report assessing immune markers in longitudinal biopsies demonstrated the importance of markers in early on-treatment samples and the non-predictive results of pre-treatment specimens^[48]^. Assessment of the markers of immune system activation should be supplemented by analysis of tumor cell death, activation of DNA damage response and tumor cell signaling pathways, employing successive biopsies.

Nevertheless, the significant clinical activity of immunochemotherapy has been documented by several meta-analyses. For example, a study covering a total of 4322 patients with advanced metastatic NSCLC who were treated with immune checkpoint inhibitors in combination with chemotherapy for first-line treatment or chemotherapy alone showed pooled HRs for OS and PFS of 0.74 (*P* = 0.0007) and 0.62 (*P* = 0.00001) in favor of the combination treatment^[49]^. Similarly, a meta-analysis based on 12 phase III studies with 9236 metastatic NSCLC patients reported a consistently higher efficacy of the combination of chemotherapy with either pembrolizumab or atezolizumab compared to chemotherapy alone^[50]^. Although checkpoint inhibitors plus chemotherapy were found to be correlated with prolonged OS, compared with chemotherapy alone (HR = 0.74, *P* = 0.0002) in an analysis comprising 2978 NSCLC patients, significant heterogeneity was revealed between trials and according to the specific checkpoint inhibitor applied and the degree of PD-1/PD-L1 expression^[51]^.

## Discussion

Conventional cytotoxic cancer chemotherapy is often immunosuppressive and associated with drug resistance and tumor regrowth after a short period of tumor shrinkage^[52]^. However, certain cytotoxic cancer chemotherapeutic drugs can kill tumor cells by an immunogenic cell death pathway, which activates robust innate and adaptive antitumor immune responses and has the potential to greatly increase the efficacy of chemotherapy. Early clinical trial data on checkpoint inhibitor combinations showed promising activity, and rapidly emerging phase III data have led to the approval of some checkpoint inhibitor combinations for first-line treatment of metastatic NSCLC^[53]^. There is therefore a great need for a systematic analysis to guide the clinical use of checkpoint inhibitor combinations. Immune checkpoint inhibitors (ICIs) have recently revolutionized cancer treatment, providing unprecedented clinical benefits, but therapy resistance can affect up to two-thirds of patients receiving ICIs^[54]^. Conventional cancer treatments, including cytotoxic chemotherapy, radiation therapy, and targeted therapy, have immunomodulatory effects in addition to direct cancer cell-killing activities.

The interaction of chemotherapy and immune checkpoint inhibitors has been tested for formal criteria of synergism^[55]^. The frontline ICI phase II/III trials in advanced NSCLC were checked for ORRs and AEs of combinations and of individual drugs. The calculations suggest an enhanced efficacy that is less than additive for the immunochemotherapy combinations since the actual ORR and AE were less than the expected additive effect. The interplay between chemotherapy and immunotherapy is unclear which impedes the design of an optimal combination strategy^[56]^. Each chemotherapy agent has unique effects on tumor cells and immune responses at different time schedules and the antitumor efficacy depends on the appropriate combination as well as the sequence and scheduling of the immunochemotherapeutic regimen^[53]^. To date, chemotherapy and immunotherapy are administered concurrently in most clinical trials disregarding the impact of sequencing on antitumor immunity. Since chemotherapy can cause immunogenic cell death that induces T cell priming, the maximal efficacy of immunotherapy should be expected after prior chemotherapy.

Preclinical and clinical studies showed controversial results with respect to the sequence of chemoimmunotherapy. In a phase II study investigating carboplatin/paclitaxel in combination with concurrent or sequenced ipilimumab in small-cell lung cancer (SCLC), sequenced treatment was associated with improved PFS^[57,58]^. In this phased regimen, paclitaxel/carboplatin alone was administered followed by ipilimumab/paclitaxel/carboplatin, which causes the release of neoantigens to precede ipilimumab exposure. In contrast, concurrent treatment with ipilimumab and chemotherapy was superior to sequential therapy in a mesothelioma model^[38]^. However, in cases of lung cancer and melanoma, chemotherapy administered after immunotherapy achieved successful clinical responses^[59,60]^. The ORR for chemotherapy administered after immunotherapy in NSCLC was significantly higher than that for chemotherapy before immunotherapy (53.4% *vs*. 34.9%)^[59]^. These findings point to the sensitization of tumors by anti-PD-1/PD-L1 inhibitors to a subsequent chemotherapy. For two cases of nivolumab-refractory lung cancer, subsequent chemotherapy achieved rapid regression^[60]^. Concerning immunochemotherapy, inhibition of PD-1 on CD8^+^-tumor-infiltrating lymphocytes restored cytokine secretion and T cell proliferation and rendered tumor cells more sensitive to chemotherapy^[61]^.

In metastatic melanoma patients who progressed after anti-PD1 monotherapy, immunochemotherapy induced a marked clinical response, with an ORR of 65% and a CR of 25%^[62]^. Responding patients exhibited a novel subset of CD8+ T cells (CX3CR1+) that survive chemotherapy with intact CTL functions^[63]^. This subset of effector T cells shows low proliferation and the ability to efflux chemotherapy drugs via the ABCB1 transporter and may be used as a biomarker in monitoring and predicting clinical response to chemoimmunotherapy. The scheduling and timing of chemotherapy in CIT are also critical for achieving clinical success. Some chemotherapy drugs work in a cell cycle-specific manner, suggesting that their direct tumor-killing activities and immunomodulatory effects can be influenced by the schedule of drug administration^[64,65]^. However, these results are based on retrospective findings on small and highly selected patient populations. More investigations are needed to define the optimal sequencing of standard chemotherapy and PD-1/PD-L1 inhibitors^[66,67]^.

## Conclusion

The approach of providing immunochemotherapy to every patient may not necessarily be an ideal solution^[68]^. Those with higher PD-L1 TPS will experience higher toxicity due to chemotherapy, which is about twice as profound as under immunotherapy alone, and patients with lower PD-L1 expression having combination treatment may receive costly and unnecessary immunotherapy. Single-agent pembrolizumab treatment is the standard first-line therapy for patients with PD-L1 ≥ 50, but immunochemotherapy for unselected patients should be replaced by precision medicine decision provided predictive biomarkers are identified.

## Declarations

### Copyright

© The Author(s) 2020.
